# Dermal Lymphatic Carcinomatosis as a Cutaneous Metastasis From Epidermal Growth Factor Receptor-Positive Lung Adenocarcinoma: A Case Report

**DOI:** 10.7759/cureus.99152

**Published:** 2025-12-13

**Authors:** Sarah A Hemelt, Jonathan M Joseph, Nicholas Culotta

**Affiliations:** 1 Department of Dermatology, Louisiana State University Health New Orleans School of Medicine, New Orleans, USA

**Keywords:** cutaneous metastasis, dermal lymphatic carcinomatosis, egfr-positive lung cancer, lung adenocarcinoma, metastatic carcinoma

## Abstract

Cutaneous metastases are an uncommon but clinically significant manifestation of internal malignancies. We present a rare case of dermal lymphatic carcinomatosis arising from epidermal growth factor receptor (EGFR)-positive lung adenocarcinoma, a presentation that may closely mimic inflammatory dermatoses and indicate therapeutic resistance or disease progression. We describe the clinical course of a 65-year-old woman who developed a violaceous cutaneous metastatic lesion beneath the left breast during treatment for metastatic lung adenocarcinoma. This case highlights the importance of recognizing cutaneous signs as indicators of disease progression and emphasizes the diagnostic challenges and prognostic implications of cutaneous metastases in lung cancer.

## Introduction

Cutaneous metastases are defined as the spread of malignant cells from internal organs to the skin and represent an important but relatively uncommon clinical finding occurring in up to 10% of malignancies [1-2]. Their presence often reflects advanced systemic disease and is associated with a worse prognosis. Clinically, skin metastases may appear as nodules, plaques, or inflammatory eruptions, and because these appearances frequently mimic benign dermatologic conditions, they can pose diagnostic challenges for clinicians [1-2].

Lung carcinomas account for around 9% of cutaneous metastases, with lung adenocarcinoma reported among the most frequent histologic subtypes to involve skin [3-4]. Dermal lymphatic carcinomatosis describes tumor infiltration of dermal lymphatic channels producing erythematous, edematous, or indurated changes that can resemble cellulitis or inflammatory breast carcinoma. This pattern of infiltration is an important, but relatively uncommon subset of cutaneous metastases [1,3]. Epidermal growth factor receptor (EGFR)-positive lung adenocarcinoma refers to tumors harboring activating EGFR mutations and are typically treated with EGFR-directed tyrosine kinase inhibitors. Cutaneous metastases occurring in patients on targeted therapy can indicate disease progression or acquired therapeutic resistance [5]. These features set the context for the present case, which illustrates diagnostic and prognostic challenges posed by dermal lymphatic carcinomatosis in an EGFR-positive patient.

## Case presentation

**Figure 1:**
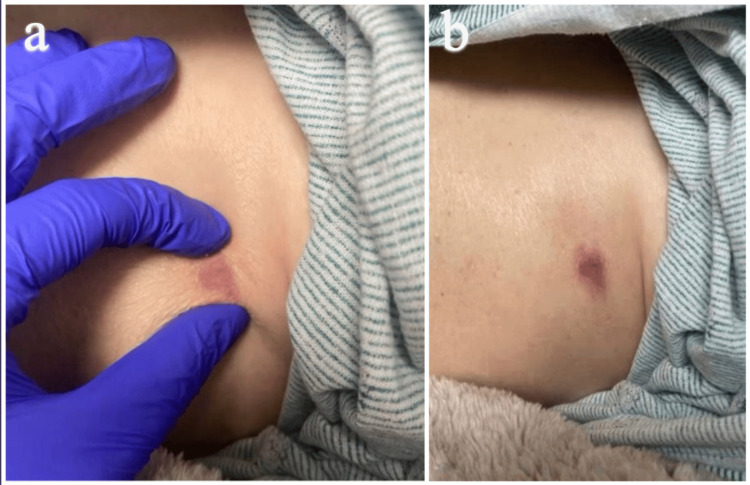
Clinical image featuring the firm, violaceous, nodular skin lesion on patient's left chest wall.

A 65-year-old Caucasian woman with a four-year history of stage IV EGFR-positive lung adenocarcinoma presented with a new firm violaceous nodule with minimal scale on the left lower chest wall beneath the breast (Figure 1). Her oncologic history was notable for metastases to the brain and bones, which had been managed with craniotomy and targeted therapy with osimertinib.

**Figure 2:**
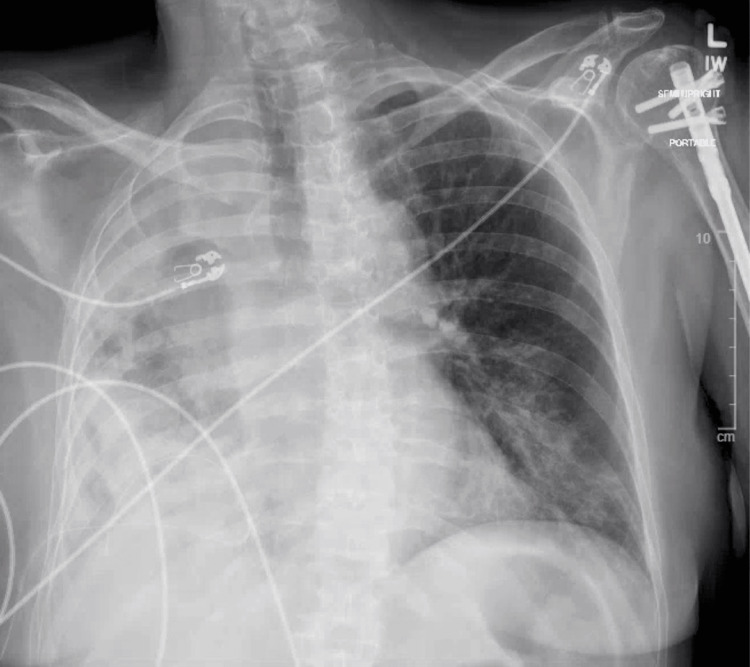
Chest X-ray demonstrating dense right-sided lung consolidation consistent with large malignant pleural effusion.

**Figure 3:**
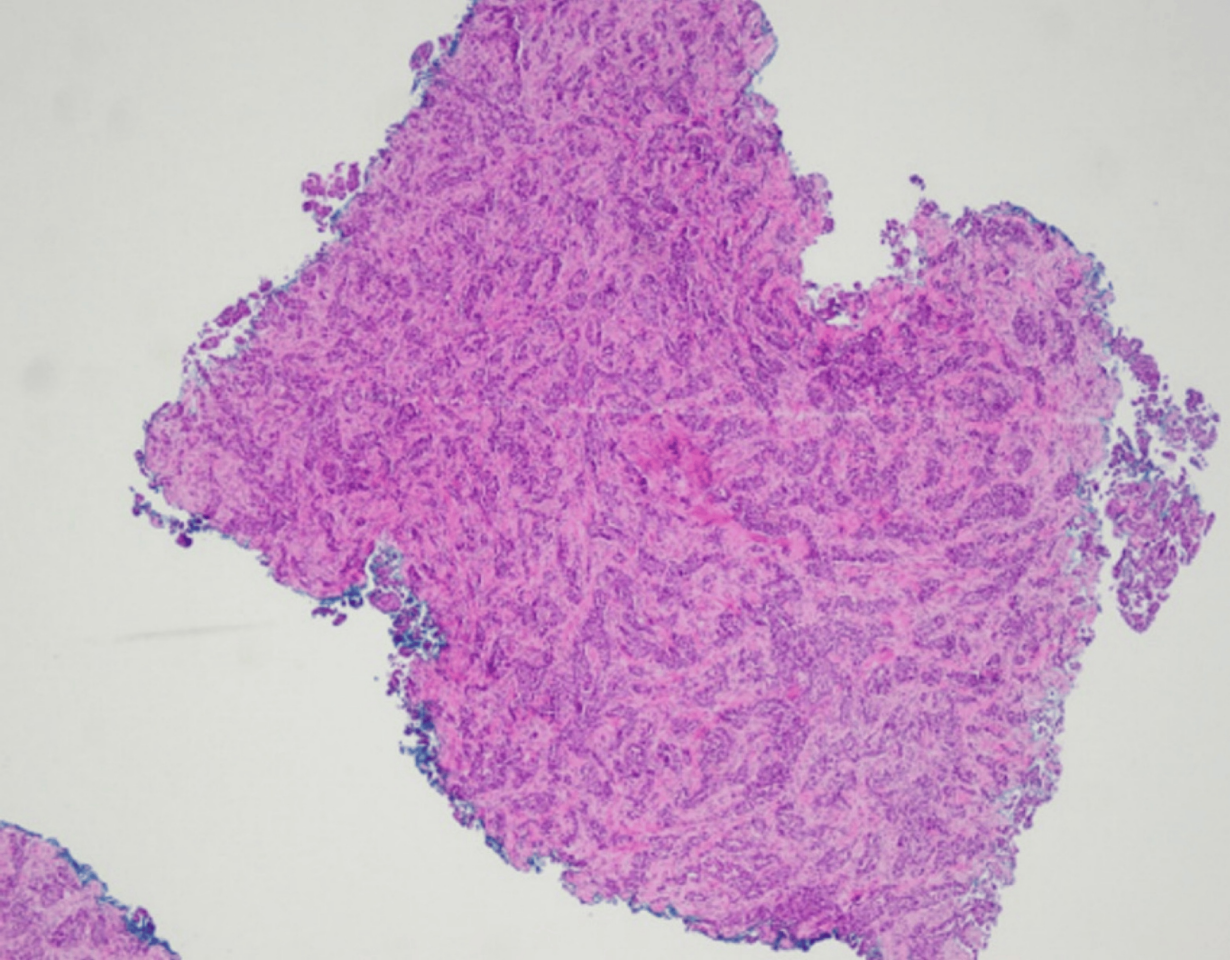
Infiltrating tumor composed of polygonal cells and luminal vacuoles arranged in nests and glandular patterns within the dermis.

**Figure 4:**
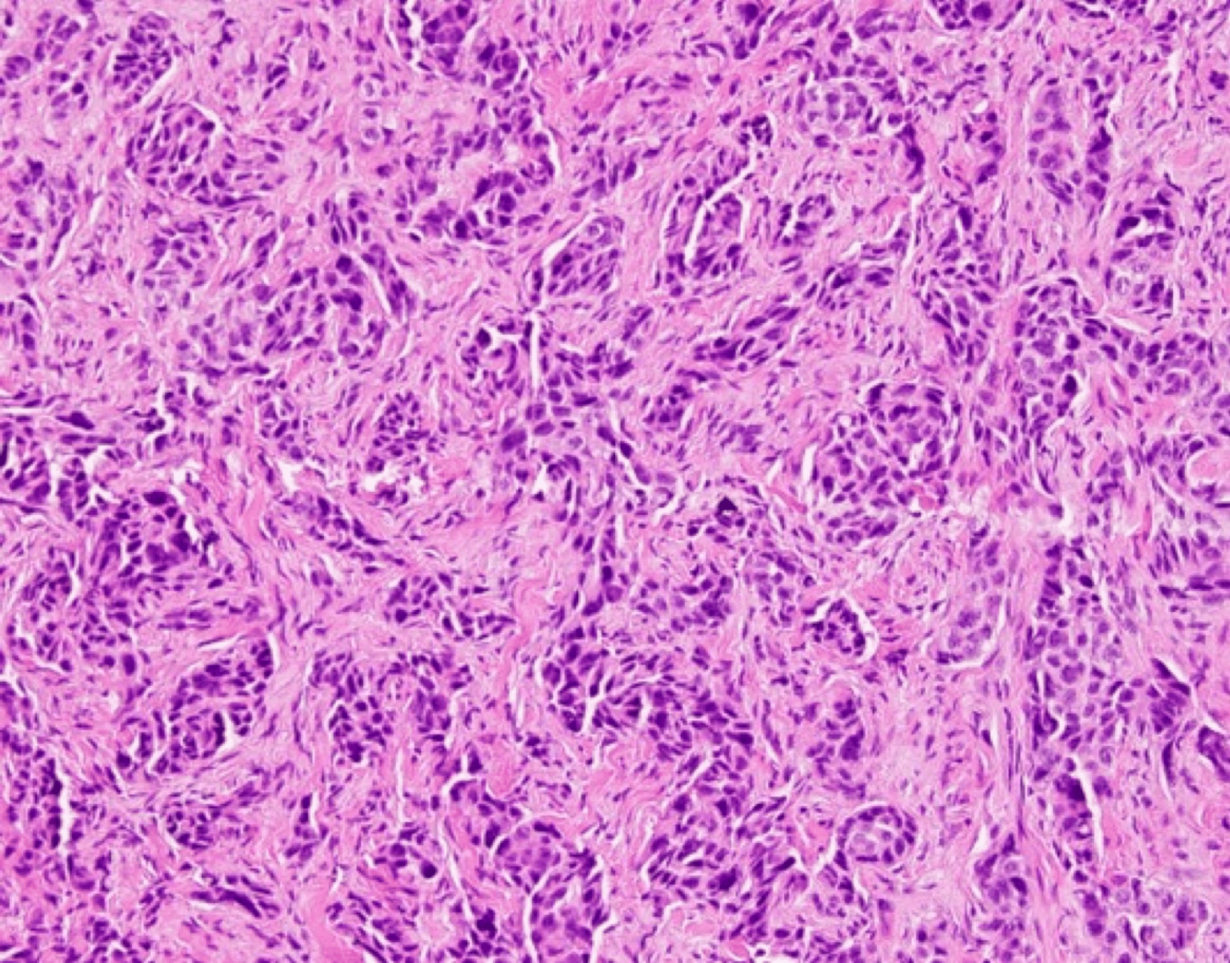
Polygonal cells with variable hyperchromatic nuclei, enlarged nucleoli, and luminal vacuoles arranged in nests and glandular patterns.

After nearly four years of ongoing treatment for lung adenocarcinoma, the course of her disease had begun to worsen, and she was experiencing progressive dyspnea. A chest radiograph indicated progression of her disease, revealing a malignant pleural effusion (Figure 2). A punch biopsy of the cutaneous lesion below the right breast was taken. Immunohistochemistry performed on the biopsy demonstrated diffuse nuclear positivity for TTF-1. The histopathology from the biopsy, along with the positive staining, is consistent with dermal lymphatic carcinomatosis secondary to metastatic adenocarcinoma of the lung (Figure 3 and Figure 4). This cutaneous lesion was a sign of systemic progression despite ongoing targeted therapy. The patient was subsequently transitioned to a chemotherapy regimen of carboplatin and pemetrexed. However, her condition deteriorated rapidly, culminating in hypoxic respiratory failure. She was transitioned to comfort care and passed away one month later.

## Discussion

Cutaneous metastases from lung adenocarcinoma typically present as rapidly growing nodules, most commonly on the chest, abdomen, and scalp [1,5]. In rare instances, malignant cells infiltrate the dermal lymphatics, leading to a presentation known as dermal lymphatic carcinomatosis. This pattern can resemble a variety of benign and inflammatory dermatoses, including cellulitis, erysipelas, lymphangitis, lymphedema, and morphea, as well as malignant conditions such as inflammatory breast carcinoma or angiosarcoma, all of which may delay accurate diagnosis and appropriate oncologic intervention [6,7].

The development of cutaneous metastases in patients with primary lung cancer is a sign of poor prognosis. Although survival outcomes may vary with mutation status, type of systemic therapy received, and disease burden, prognosis tends to be poor. Following the diagnosis of cutaneous metastases, survival rates range from four to six months in some cases [5,8]. In cases of cutaneous metastasis with dermal lymphatic involvement, the prognosis is particularly poor, with some reports describing survival of three months or less [9]. This poor survival outlook reflects the aggressive nature of metastatic lung adenocarcinoma and the limited efficacy of systemic therapies at this stage of disease progression. The emergence of cutaneous metastases may also suggest therapeutic resistance or be a sign of refractory disease, necessitating prompt evaluation of the current treatment plan [10].

Increased awareness of the importance of cutaneous metastases is crucial, as cutaneous metastases may be the first or only sign of disease progression in patients receiving targeted therapies. This highlights the importance of the role dermatologists play in the multidisciplinary care approach to those diagnosed with malignancies. Prompt biopsy and histological evaluation of any suspicious lesions, including immunohistochemical staining with markers such as TTF-1, can aid in establishing the diagnosis and confirming the pulmonary origin of the metastases [6]. Thyroid transcription factor 1, TTF-1, is a nuclear marker that is highly specific for primary lung adenocarcinoma and is thus helpful in distinguishing pulmonary from extrapulmonary metastases [6,11]. Immunohistochemical markers are often used in conjunction to improve diagnostic accuracy.

Clinicians must maintain a high index of suspicion for new or atypical cutaneous lesions. This is particularly important for patients with a current or past medical history of malignancy. Heightened clinical awareness of these lesions can lead to earlier detection, facilitate timely interventions, and improve patient-centered care through informed discussions about prognosis and treatment goals.

## Conclusions

Cutaneous metastases are an uncommon but clinically meaningful finding in patients with lung adenocarcinoma. Their presence is often indicative of widespread systemic disease and correlates with poor prognosis. Dermatologists play a critical role in early detection through biopsy and clinical suspicion. Timely diagnosis, in collaboration with oncology, can facilitate appropriate adjustments in management and inform discussions about prognosis and goals of care. Greater awareness of these cutaneous metastatic manifestations may lead to earlier interventions and improved patient-centered outcomes overall.

## References

[1] Schwartz RA (1995). Cutaneous metastatic disease. J Am Acad Dermatol.

[2] Vernemmen AI, Li X, Roemen GM (2022). Cutaneous metastases of internal malignancies: a single-institution experience. Histopathology.

[3] Molina Garrido MJ, Guillén Ponce C, Soto Martínez JL, Martínez Y Sevila C, Carrato Mena A (2006). Cutaneous metastases of lung cancer. Clin Transl Oncol.

[4] Sharma G, Kumar P, Veerwal H, Singh P, Gupta S, Dhingra V (2021). Cutaneous metastases as initial presentation of lung carcinoma. Cureus.

[5] Hidaka T, Ishii Y, Kitamura S (1996). Clinical features of skin metastasis from lung cancer. Intern Med.

[6] Alcaraz I, Cerroni L, Rütten A, Kutzner H, Requena L (2012). Cutaneous metastases from internal malignancies: a clinicopathologic and immunohistochemical review. Am J Dermatopathol.

[7] Lookingbill DP, Spangler N, Helm KF (1993). Cutaneous metastases in patients with metastatic carcinoma: a retrospective study of 4020 patients. J Am Acad Dermatol.

[8] Betlloch-Mas I, Soriano-García T, Boira I, Palazón JC, Juan-Carpena G, Sancho-Chust JN, Chiner E (2021). Cutaneous metastases of solid tumors: demographic, clinical and survival characteristics. Cureus.

[9] Prat L, Chouaid C, Kettaneh A, Fardet L (2013). Cutaneous lymphangitis carcinomatosa in a patient with lung adenocarcinoma: case report and literature review. Lung Cancer.

[10] Terashima T, Kanazawa M (1994). Lung cancer with skin metastasis. Chest.

[11] Forest F, Laville D, Habougit C (2021). Histopathological and molecular profiling of lung adenocarcinoma skin metastases reveals specific features. Histopathology.

